# Portrait of Boredom Among Athletes and Its Implications in Sports Management: A Multi-Method Approach

**DOI:** 10.3389/fpsyg.2020.00831

**Published:** 2020-05-26

**Authors:** Franklin Velasco, Rafael Jorda

**Affiliations:** Department of Marketing, Universidad San Francisco de Quito, Quito, Ecuador

**Keywords:** boredom, sports management, athlete performance, overconsumption, brand loyalty, emotions

## Abstract

There is a common misconception that elite athletes enjoy their sports activities so much that they cannot feel bored. However, this research reveals that boredom is a prevalent emotion among professional, amateur, and college athletes that impacts their performance, brand preferences, and overconsumption behaviors. This investigation relies on a multi-method approach. Qualitative data were collected through interviewing athletes (*n* = 123), and the critical incident technique was used to record factual boredom incidents. Quantitative data were collected through a survey and analyzed using hierarchical regression models. The purpose of the survey was to evaluate athletes’ proneness to boredom and then present a typical sports consumption scenario in which athletes’ brand preferences and overconsumption behaviors were captured. Overall findings from this research indicate that episodes of boredom are common among athletes when they engage in repetitive tasks (34.8%); negative mood is anticipated (16.9%); teammates show a lack of interest and seriousness (15.7%); they must endure periods of waiting (13.5%); there is a lack of competitiveness and goal-setting (10.1%); there is a lack of participation in activities (4.5%); there is a lack of empathy with teammates and coaches (3.3%); and there are infrastructure issues (1.1%). Furthermore, this study presents evidence that boredom negatively impacts athletes’ performance (β = −0.41). Then, in a specific sports consumption scenario that uses sports drinks, this study finds that a more boredom-prone athlete has a higher chance of purchasing different brands of the same product (β = 0.37) and engaging in overconsumption behaviors (β = 0.44). The relationships among boredom, performance, variety seeking, and impulse buying are congruent with previous research on boredom. This research discusses several sports management implications and presents recommendations from coaches on how to cope with athletes’ boredom.

## Introduction

Boredom has usually been described as a negative emotion experienced in relation to monotonous types of activities (Halbesleben and Buckley, 2004). Fisherl (1993) depicted boredom as a universal emotion that nearly everyone experiences, irrespective of the nature of their job. Since elite athletes can be described as having a full-time job and are considered employees of their respective teams, institutions, and leagues (Tainsky and Babiak, 2011), it is reasonable to expect that they experience boredom. Sports managers and fans might see this as counterintuitive, as they might perceive athletes’ jobs as simple tasks in which competitiveness, playfulness, and excitement are more prevalent emotions than boredom.

Although research interest in boredom is increasing, there is much more to be explored about this influential negative emotion (Tze et al., 2016). Undeniably, a shortcoming of boredom studies in a sports context is that it reports mixed findings. For example, Chin et al. (2017) reported that adults consider practicing sports one of their least boring activities. However, other studies have argued that boredom undermine school athletes’ motivation to practice sports (Duda and Nicholls, 1992) and decreases the intention to persist with physical activity (Pulido et al., 2014). Because boredom in society is incremental (Mael and Jex, 2015), and it seems that boredom is more prevalent in professional athletes (Atousa and Sheykhshabani, 2012), it is critical to explore athletes’ experiences with boredom and evaluate its consequences from a sports management perspective. The present research fills this gap by presenting a series of boredom incidents in elite athletes, a classification of these incidents into substantive categories, and a report on the consequences of boredom in sports consumption scenarios.

Organizational behavior literature has identified boredom as a relevant stressor variable present in different types of jobs and activities (Loukidou et al., 2009; Bruursema et al., 2011; Schaufeli and Salanova, 2014; Harju et al., 2018). Boredom refers to an unpleasant feeling during individuals that causes a lack of interest in a current activity, pensiveness, and difficulty concentrating on a task (Game, 2007). Pekrun (2006) conceptualized boredom as a multi-dimension negative emotion that includes several components: affective components (i.e., unpleasantness and negative attitude toward an activity), cognitive components (i.e., time distortion perceptions, reduced attention, and constraint), physiological components (i.e., low arousal), non-verbal communication components (i.e., pensiveness and facial and postural expressions), and motivational components (i.e., strong efforts to overcome the low arousal state). The state of boredom “makes people feel like they are emotionally trapped and at the same time contributes to senses of loss of value, significance, and meaning” (Elpidorou, 2014, p. 2).

Bored individuals have been found to be less persistent and more gregarious (Leong and Schneller, 1993), impulsive (Leong and Schneller, 1993; Moynihan et al., 2017), and attracted to sensation seeking (Kass and Vodanovich, 1990). Following these findings, it is possible to anticipate that when athletes feel bored, the sensations of being restless, inattentive, and distant might harm their performance. Moreover, because the state of boredom makes people feel under-stimulated, athletes might cope with boredom by engaging in behaviors such as overconsumption (i.e., buying more sport products than needed) and variety seeking (e.g., changing their loyalty among sports drink brands). In this research we have focused on investigating these effects of boredom and on trying to identify with detail athletes’ boredom incidents using a multi-method approach.

This research presents two studies that aimed to expand the understanding of boredom in a sports management context. The first study presented a taxonomy of boredom episodes. Findings from this study reveal that boredom is prevalent among athletes. The second study investigated more deeply boredom’s influence on athletes’ performance, brand preferences, and overconsumption behaviors. Findings from this study reveal that boredom negatively influences athletes’ performance and shapes their brand choices (variety seeking) and consumption behaviors (overconsumption).

The present research aimed to examine what aspects of being an elite athlete at the professional, amateur, or college level trigger boredom and how this negative emotion influences athletes’ performance, attitudes, and behaviors. Furthermore, the purpose of this investigation was the identification of boredom incidents among elite athletes. In addition, this research discusses several practical implications for coaches and sports managers about dealing with athletes’ boredom. Our findings allow sports management institutions to rethink their tactics and psychological approaches to help athletes cope with boredom episodes. In addition to this, our findings provide insight for brand managers in terms of designing promotional strategies.

## Materials and Methods

The research approach of our investigation combined qualitative and quantitative methods. Since this paper aimed to expand the understanding of boredom in a sports management context, Study 1 used the critical incident technique (CIT; Flanagan, 1954; Gremler, 2004) as a tool to detail boredom experiences in athletes. Study 2 relied on survey data to examine boredom’s influence on athletes’ performance, brand preferences, and overconsumption behaviors. Next, we have presented the sample characteristics, measures, procedures, and data analysis descriptions of our studies.

### Participants

The sample strategy included several rules and criteria to recruit participants. First, we invited a list of coaches (*n* = 16; Mean_age_ = 43.58, SD = 9.58; Mean_tenure_ = 17.65, SD = 9.64) to share their player rosters and participate in a study about athletes’ emotions.

A total sample of 134 elite athletes from Ecuador participated in this study. The participants were between 16 and 66 years old (Mean_age_ = 22.56, SD = 6.05). In terms of sex, 64.2% were males (Mean_age_ = 23.26, SD = 7.19) and 35.8% were females (Mean_age_ = 21.31, SD = 2.79). In terms of athletes’ level, 80.5% compete at college level and 19.5% at professional level.

The distribution of the sample by sport discipline was men’s soccer (29.1%); volleyball (9.7%); basketball (9.0%); aerobics (8.2%); women’s soccer (7.5%); taekwondo (6.7%); jiu-jitsu (6%); table tennis (6.0%); tennis (4.5%); track and field (3.7%); triathlon (3.7%); gymnastics (3.0%); and weightlifting (3.0%). This diverse sample of sport disciplines was intentionally planned to achieve a higher degree of external validity for the study’s findings and to use as a control variable in the regression analysis.

### Measures

Because the purpose of this research was to identify how boredom manifests in athletes and to understand the consequences of episodes of boredom in athletes’ consumption behaviors, we relied on a multi-method research approach.

For Study 1, which focused on the identification of boredom episodes, we relied on the CIT^1^ to collect actual incidents and classify them into categories. For the purpose of this study, these critical incidents were defined as events or series of events when athletes started feeling bored when practicing or competing. The interview started by asking athletes to recall the last time they felt bored when they were either practicing or competing in their sport. We further asked them to describe this situation with as many details as possible. For example, “What were your thoughts and feelings at that particular moment?”; “What do you think was the root cause for you feeling bored?”; “To what do you attribute this feeling?”; and “To you what kind of context generated feeling bored?” The average time per interview for the boredom’s CIT was 16.38 min.

The second part of the study, Study 2, consisted of collecting quantitative data via a survey after the interview. The instrument included measures about boredom proneness and consumption scenarios in which we measured brand preferences for sports drinks and overconsumption behaviors. We used Beaton et al.’s (2000) backtranslation technique to translate the survey from English to Spanish. A professional translator translated the survey from Spanish to English. The two authors then simultaneously translated the survey. Then, one academic back translated the survey to English and solved discrepancies by comparing the original items with the new set of items. Authors pilot tested the questionnaire with a sample of undergraduate students. In the next paragraphs we have presented in detail each of the measures included in the instrument.

*Athletes’ boredom proneness* was measured with five items adapted from the short version of the boredom proneness scales developed by Struk et al. (2017). The initial eight-item scale was adapted by including in the original wording a few words referring to sports (e.g., practices and training). This scale was validated by the group of coaches that shared their players roster and participated in our study. Coaches decided that some items were not applicable to all sports disciplines or difficult to understand in a sports context. We have listed some examples of items that were excluded: “I often find myself in practices at loose ends, not knowing what to do”; “I find it hard to entertain myself at practices”; “In most situations, it is hard for me to find something to do or see to keep me interested”; “Much of the time, I just sit doing nothing”; and “Unless I am doing something exciting, even dangerous, I feel half-dead and dull.” The final scale resulted in five items. Participants expressed with a seven-point scale how much they agreed or disagreed with various statements: “It is difficult for me to concentrate on my training activities”; “Time always passes very slowly when I attend practices”; “Many things I have to do during practices are repetitive and monotonous”; “It takes more stimulation to get me going than most players”; and “At practices, I am bad at waiting patiently” (α = 0.70). The scale was validated with exploratory factor analysis (EFA) using principal components method. Varimax rotation was used since evidence suggests that this rotation method is preferred over other types of rotations for identifying a simple structure (Tabachnick et al., 2007; DeVellis, 2016). The analysis indicate a one-factor solution that include all final five items of the scale and showing loadings higher than 0.4. Appendix A in the Supplementary Material report the factor loadings.

*Overall performance* was measured by a single item. This measure follows a scheme designed by the coaches included in our sample who created an overall measure of athletes’ performance. Future performance was defined as likelihood of achieving international success. In this way, the scale conceptualizes top performers as those athletes’ that have strong probabilities of achieving international success versus low performers conceptualized as athletes’ demonstrating irregular performance levels that will not achieve even local success. Therefore, coaches responded to the question, “Overall, how would you evaluate this player in terms of his/her future performance?” using a six-point scale: “5 = the player has the potential to obtain, in the near future, an international achievement”; “4 = the player has the potential to obtain, in the near future, a local achievement”; “3 = the player will maintain having an outstanding performance at the team and personal levels”; “2 = the player will demonstrate greater effort and will distinguish from other teammates”; “1 = the player will maintain a regular performance”; and “0 = the player will demonstrate an irregular performance with highs and lows.”

*Brand preference* was measured by the athletes’ number of brand choices in a sports drinks’ consumption scenario. This operationalization allowed us to capture athletes’ variety-seeking behaviors, as a greater number of brands selected signaled lower brand loyalty and stronger variety seeking. Athletes were asked to imagine they were planning to buy 10 sports drinks for their weekly training activities based on a free choice of brands. Five options of brands familiar to athletes were presented in a list (i.e., Gatorade^TM^, Powerade^TM^, Sporade^TM^, and Profit^TM^), including an option for participants to write in a brand that was not on the list. With this they were presented a statement that brands did not differ as to price. Athletes answered this question by entering the number of bottles of each brand they wanted to purchase. The number of different brands they chose became our second dependent variable.

The *overconsumption* variable was operationalized as a measurement within the same consumption context described above. After athletes completed their brand preferences for sports drinks, they were asked to imagine there was a promotion going on at the time of purchase. Since they just bought 10 bottles, athletes would receive a “big discount” if they opted to buy more. Athletes then entered the number of additional sports drinks they wanted to add to their purchase in a range between 0 and 10. This number became our third dependent variable.

### Procedure

The Institutional Review Board approved this study’s procedure. All participants voluntarily agreed to participate in the study and registered their consent. Before the data collection took place, a meeting with managers and coaches was held to inform them about the study’s procedure and purposes. Further, coaches and athletes were guaranteed the confidentiality of their data and personal information.

First, we asked coaches to share their player rosters and asked them to provide an overall performance score for each of their players, a measure that served as one of our dependent variables. Second, coaches were invited to fill out a short online survey prompting them to describe an episode of boredom they had experienced recently when working with their athletes. This question tracked the CIT by collecting factual boredom incidents from coaches. Its purpose was twofold: (1) to examine and categorize boredom incidents among athletes and (2) to present a list of coaches’ recommendations as to the tactics sports managers could implement to reduce athletes’ boredom. Appendix B in the Supplementary Material presents the illustrative quotes from coaches describing boredom incidents.

Third, using the list of athletes that the coaches provided, we randomly invited players to participate in an interview in which we applied the CIT (e.g., record boredom incidents) and to complete a short survey that included our set of dependent measures and some demographic information. Four research assistants who were trained with the CIT and the instrument designed for this study helped in the data collection process over a period of 2 months.

### Data Analysis

The authors and three independent judges completed the CIT content analysis by coding the incidents into categories. The process consisted of repeated reading of the data and identifying similarities among the athletes’ responses. Disagreements were resolved through discussions, and final inter-judge reliability was 92%.

The second part of the data analysis was the assessment of the survey data. To understand how boredom influences athletes’ performance, brand preferences, and overconsumption behaviors, we relied on multiple linear regression analysis using SPSS software (IBM SPSS, Version 24.0). Hierarchical linear regressions, with athletes’ boredom proneness scores as predictors, were performed for each of the three dependent variables. Through a second step, the control variables (i.e., athletes’ gender, age, sport discipline type, and tenure) were entered into each regression model to observe differences in the effects. The statistical tests of each predictor were two-tailed at an alpha level of 0.05. To control for a type 1 error, we reported standardized regression coefficients and *p*-values calculated for the non-parametric correlation analysis ensuing the sequential Bonferroni–Holm practice (Holm, 1979). Descriptive statistics for the variables included in each of the models and correlations were calculated. Table 1 shows these calculations.

**TABLE 1:** Descriptive statistics and correlations.

Variable	Mean	SD	1	2	3	4
1. Athletes’ boredom proneness	3.32	1.33				
2. Overall performance	3.07	1.57	−0.40**			
3. Brand preferences	2.28	1.25	0.38**	−0.08^ns^		
4. Overconsumption	4.91	3.33	0.47**	−0.17*	0.14	

*p < 0.10; **p < 0.01. ns, not statistically significant.

## Results

### Study 1 Boredom Incidents Among Elite Athletes

The contribution of Study 1 involves identifying boredom incidents among athletes and classifying them into theoretical categories. As discussed before, the authors followed a systematic process to perform the content analysis of the incidents reported by the athletes. This systematic process incorporated an extensive literature review into the psychological determinants of boredom. During the content analysis, commonalities were found between boredom incidents in the study and theoretical categories from previous boredom literature. Table 2 illustrates the results of the content analysis.

**TABLE 2:** Percentage of athletes’ boredom incidents falling into each content category.

Category name	Illustrative quotes	Percentage of total
1. Monotonous and repetitive activities	The last time I felt bored in training was when the workouts were very repetitive, doing the same tasks every Monday of every week, even the next days. **_taekwondo athlete_**	34.8
	I felt bored in training because the workout and routine did not vary, and everything was very repetitive. This often tires me because doing the same thing does not help much. **_basketball player_**	
2. Anticipated negative mood	Currently I am feeling very tired and sleepy when attending the morning training sessions. That is why I feel bored. **_soccer player_**	16.9
	I was working out with four other girls in a series of exercises we needed to complete, but I was mad. So, I wanted to leave the practice because I was feeling frustrated and bored at the same time. **_gymnastics athlete_**	
3. Teammates’ lack of motivation seriousness	I feel bored when no one shows up to train or start doing other things the coach assigns to us. **_volleyball player_**	15.7
	Basically, I get bored because there are no people at my same level. **_tennis player_**	
4. Waiting	I was waiting for the next game, and since I did not have anything to do until the game starts, I was feeling bored and lazy. **_tennis table player_**	13.5
	I am always bored when our coach had us sitting down doing nothing while training other girls from another category. **_women’s soccer player_**	
5. Lack of competitiveness and challenges	I am used to getting bored when there is no upcoming competition. The last time was during summertime in which I was in great physical and mental condition, but there was no budget to compete and training without competing is frustrating especially in contact type of sports. **_jiu–jitsu athlete_**	10.1
	The last time I was bored was because I could not understand the function of the exercises, they were too simple, and just thought about when practice ends. **_gymnastics athlete_**	
6. Lack of participation in activities	The truth is that I really like practices, but I get bored when one group of girls is more actively involved in training while the others are just passing the balls. If we were having a more active task, the training would be more pleasant. **_women’s soccer player_**	4.5
	I am bored at games that I am not playing, and the team is winning easily. My teammates play very well, and we were ahead on the scoreboard by several points, so I was just watching the game.**_ volleyball player_**	
7. Lack of empathy with teammates and coaches	We recently switch to a new coach, but I do not like him. I still miss my old coach, so I felt frustrated and bored at the same time. **_triathlon athlete_**	3.3
	When other players do not show a good attitude and respect, I start thinking about other things I have to do or listen to music to entertain myself while playing. **_men’s soccer player_**	
8. Infrastructure issues	The last time I was bored was about a year ago. We had workouts without the necessary equipment and uniforms. In those moments, it felt like I simply did not want to continue being part of the team. This caused the whole season to become boring.**_ tennis table player_**	1.1

The first category of boredom incidents among athletes is monotonous/repetitive activities, which make up 34.8% of the total incidents. Athletes feel bored during practices when they are forced to do repetitive workouts and exercises that are similar to recent past experiences and when routines do not vary. Quotes related to this category included, “What happens is that some workouts are similar and repetitive, which is why sometimes it is a bit boring” and “Sometimes I start thinking that I could be doing other activities or even taking a nap when the coach asked us to repeat the exercises.”

According to the content analysis of boredom incidents, the second category of boredom incidents represent 16.9% of the total incidents. These boredom episodes are related to anticipated negative mood. When athletes feel frustrated, apathetic, depressed, or pessimistic, feelings of boredom are activated. There are several examples of athletes’ responses related to this category: “There are times that due to university’s workload, like homework and projects, I do not feel excitement to train and everything seems boring to me” and “Boredom happens when I feel frustrated about the other things I have to do related to homework, my job, and upcoming exams.”

The third category is related to circumstances when athletes believe their teammates are not involved or simply are absent from practices, feelings of boredom appear. A noteworthy amount of boredom incidents (15.7%) are related to athletes’ perceptions regarding teammates’ lack of motivation/seriousness. This is an example of a quote that belongs to this category: “I felt bored when my colleagues showed no interest in the workout, and they do not take it seriously.”

In this study, 13.5% of boredom incidents fell into the fourth category of waiting experiences. The following quote exemplifies statements in this category: “The other day I felt bored when the trainer was focused on certain players working on their mistakes, while the others were just watching them. Because my teammates continued doing mistakes, we just had to lay down and keep waiting.”

The CIT content analysis identified that 10.1% of boredom incidents are linked to a lack of competitiveness and challenges. Elite athletes develop their skills within a competitive context. One athlete mentioned that “the last time I felt bored was about 8 months ago because at that time I did not have a goal in mind. I felt there was no direction, or something to aim for. I could not find myself, as if everything was against me. I questioned what I was doing. Then, I started to have a clear mind with specific goals and my performance started to improve.”

Several of the boredom incidents (4.5%) that athletes reported include instances when they are not actively participating in sports activities. These activities include tasks in which they slow down or cool down and activities they perceive as unmeaningful. Examples include when a team is winning easily, when one group of athletes is more actively participating while others are not, or when they engage in secondary activities (e.g., picking up balls or warming up). The following quote refers to a secondary activity: “The last time I felt bored was when I had to pick up balls for a long time and I started to cool down. The moment I begin to cool down, I do not have the same motivation. I feel that cooling down is not good because it takes time to warm up again and the excitement goes away.”

Athletes’ boredom incidents in the study reveal a lack of empathy with teammates and coaches is a distinctive cause of boredom. This category corresponds to 3.3% of the total boredom incidents and reflects athletes’ subjective feelings on comfort with or distance from others. An example within this category is the following quote: “Yesterday we were in a game, but I just stopped playing because I did not feel comfortable with the people I was playing against and started feeling bored.”

The last category is associated with how sports infrastructure issues generate boredom in athletes. In the study, 1.1% of the total number of incidents are associated with *sportscape* issues. Sportscape is related to the physical environment and the facility management (e.g., facility aesthetics, equipment maintenance, facility comfort, etc.) of sports institutions or teams. In essence, the role of the sportscape is to provide a pleasant atmosphere for both, customers, and employees. When athletes perceive that the sports organization does not adequately maintain fields, equipment, and infrastructure, they develop a sense of frustration, which in turn triggers boredom. A quote that exemplifies what athletes said in relation to the sportscape and its link with boredom is the following: “I felt bored while training when the university courts are in very bad condition and you could not play at high level. The ball bounced and went anywhere. That bores me.”

### Study 2 Athletes’ Boredom Proneness Effects in Athletes’ Performance, Brand Preferences, and Overconsumption Behaviors

In this section, we have presented the results of the collection of survey data from athletes. The purpose of Study 2 is to provide a deeper understanding of boredom’s influence on athletes’ performance, brand preferences, and overconsumption behaviors. In addition, the findings of Study 2 present initial evidence on how boredom affects relevant variables related to sport management. Therefore, three multiple regression models were constructed. Each regression model utilized athletes’ boredom proneness as predictor. In the second step, control variables were entered into the model. Multi-collinearity was not a factor on any of the regression models. None of the variables presented a variance inflation factor greater than 1.24.

Table 3a presents the results of the hierarchical regression model that tests the influence of athletes’ boredom proneness on athletes’ overall performance. The final model, model 2, explains 23% of the variance of athletes’ performance and produced a significant *F*-score_(df2=4)_ = 7.48. This result demonstrates that boredom impacts athletes’ performance. The standardized regression coefficient for athletes’ boredom proneness, β = −0.41, was statistically significant (*t* = −5.11; *p* < 0.001), confirming that boredom negatively influences athletes’ performance.

**TABLE 3a:** Athletes’ performance regression analysis.

Model	Predictor	*b*	SE	β	*t*	*p-*value	VIF
1	Constant	4.81	0.35		13.78	0.00	
	Athletes’ boredom proneness	–0.51	0.09	–0.42	–5.27	0.00	1.00
2	Constant	–95.49	45.63		–2.09	0.00	
	Athletes’ boredom proneness	–0.49	0.09	–0.41	–5.11	0.00	1.03
	Athletes’ gender	0.06	0.26	0.02	0.24	0.81	1.06
	Tenure	0.02	0.02	0.05	0.58	0.56	1.23
	Age	0.05	0.02	0.19	2.19	0.03	1.24
	Type of sport discipline	0.04	0.03	0.13	1.58	0.12	1.05

*Model 1: R^2^ = 0.18; R-square change = 0.18; F-value = 27.76, p < 0.001. Model 2: R^2^ = 0.23; R^2^ change = 0.05; F-value = 7.48, p < 0.001.*

Considering the relationship between athletes’ boredom proneness and brand preferences, the results from the second regression model support the notion that when athletes feel bored, they will seek variety in the brands they choose. Table 3b presents the regression results that indicate that boredom leads to the extension of brand preferences.

**TABLE 3b:** Brand preferences regression analysis.

Model	Predictor	*b*	SE	β	*t*	*p-*value	VIF
1	Constant	1.12	0.28		3.95	0.00	
	Athletes’ boredom proneness	0.35	0.08	0.36	4.41	0.00	1.00
2	Constant	42.62	37.07		1.15	0.25	
	Athletes’ boredom proneness	0.36	0.08	0.37	4.57	0.00	1.03
	Athletes’ gender	0.29	0.21	0.11	1.34	0.18	1.06
	Tenure	–0.04	0.02	–0.19	–2.18	0.03	1.23
	Age	–0.02	0.02	–0.10	–1.12	0.26	1.24
	Type of sport discipline	0.03	0.02	0.12	1.51	0.13	1.05

*Model 1: R^2^ = 0.13; R^2^ change = 0.13; F-value = 19.46, p < 0.001. Model 2: R^2^ = 0.18; R^2^ change = 0.05; F-value = 5.72, p < 0.001.*

The regression model shows that 18% of the variance in brand preference is explained by athletes’ boredom proneness. The final model, Model 2, produced a significant *F*-score_(df2=4)_ = 5.72. The standardized regression coefficient for athletes’ boredom, β = 0.37, was statistically significant (*t* = 4.57; *p* < 0.001), suggesting that boredom increases athletes’ likeliness of choosing different brands among a product category.

On a separate note, the regression model also indicated that how much experience the athlete has in practicing a sport plays a significant role in brand preferences. Tenure shows a negative and significant regression coefficient. Thus, the more experienced athletes demonstrate lower variety-seeking behaviors in their brand choices for sports drinks.

The third regression model included athletes’ boredom proneness as a predictor of overconsumption. For this study’s purposes, overconsumption was operationalized as how many extra bottles of a sports drink participants chose to purchase after they were granted a discount due to the 10 bottles they previously purchased. This consumption context and measure allowed us to capture athletes’ overconsumption behavior as a consequence of feeling bored.

Table 3c presents the regression results. The regression model, Model 2, produced a significant *F*-score_(df2=4)_ = 8.35. In this case, athletes’ boredom proneness explains 25% of the variance in overconsumption decisions made by athletes. The standardized regression coefficient for athletes’ boredom, β = 0.47, was statistically significant (*t* = 5.94; *p* < 0.001), suggesting that boredom increased athletes’ likelihood of engaging in impulse buying (e.g., overconsumption).

**TABLE 3c:** Overconsumption regression analysis.

Model	Predictor	*b*	SE	β	*t*	*p-*value	VIF
1	Constant	1.26	0.72		1.75	0.08	
	Athletes’ boredom proneness	1.11	0.19	0.44	5.63	0.00	1.00
2	Constant	–130.35	93.79		–1.39	0.16	
	Athletes’ boredom proneness	1.17	0.19	0.47	5.94	0.00	1.03
	Athletes’ gender	–0.63	0.54	–0.09	–1.17	0.24	1.06
	Tenure	0.09	0.05	0.16	1.84	0.07	1.23
	Age	0.06	0.04	0.12	1.39	0.16	1.24
	Type of sport discipline	0.10	0.05	0.15	1.85	0.07	1.05

*Model 1: R^2^ = 0.20; R^2^ change = 0.20; F-value = 31.66, p < 0.001. Model 2: R^2^ = 0.25; R^2^ change = 0.05; F-value = 8.35, p < 0.001.*

## Discussion

Although most people think that elite athletes are privileged because they have managed to make their passion their profession, the reality is different. The aim of the present research was to record incidents of boredom from athletes and examine how boredom affects athletes’ performance, attitudes, and behaviors. In doing so, this study identifies that in more than 80% of cases athletes have suffered or suffer from boredom. Results from this study include a set of psychological factors or categories that are related to incidents of boredom. Therefore, we have concluded that boredom is a prevalent emotion in elite athletes. Moreover, this study has presented evidence that boredom negatively influences athletes’ performance, increases the chances athletes engage in variety seeking behaviors, and increases the chances athletes engage in overconsumption behavioral patterns. Next, we have discussed these feelings in more depth.

### Categories of Boredom Incidents and Implications to Sports Management

Insights from the CIT highlight that feelings of boredom among athletes are triggered when: (a) they engage in monotonous tasks; (b) they feel frustration or anticipate negative moods; (c) they perceive a lack of motivation and lack of teammate involvement; (d) they must endure periods of waiting; (e) there is an absence of clean-cut sports objectives or awareness of forthcoming competitions; (f) they are not participating in the team’s activities; (g) there is a lack of empathy with coaches and other teammates; and (h) sports’ infrastructure issues cause delays or problems. These categories can be cataloged as psychological determinants for boredom in a sports context.

The abovementioned phenomena represent challenges for sports managers because they not only have to recruit and train the best athletes to increase the team’s performance, but they also must train coaches and players on how to cope with boredom in order to keep the athletes’ morale and motivation at high levels.

The most important category to consider in relation to boredom, due to the high incidence rate, is monotony and repetitive activities. Illustrative quotes from athletes suggest that coaches should vary the activities they perform in training. It is also necessary to have a flexible plan of activities with a declared working objective to avoid monotony. Monotony and boredom are very closely related (Smith, 1955). Monotony causes psychological distress (Melamed et al., 1995), results in decrements in vigilance and task performance (Cummings et al., 2016), and can be so unpleasant that people seek out pain if it is their only alternative (Bench and Lench, 2019).

Second, in order of incidence, is the anticipated negative mood of the athletes. Negative mood is caused by external factors that result in distractions, frustration, and concern in athletes. This could be related to how athletes interpret their emotions. The appraisal theory of emotions (Ellsworth and Smith, 1988; Scherer et al., 2001; Moors et al., 2013) underscores that subjective emotions are interconnected, and emotions are driven by appraisals. When an athlete is in a bad mood, negative appraisals about practices and workouts create other negative valence emotions, including boredom. According to Plutchik (2001), contempt, disgust, and anger are emotions that share components (i.e., having high control and common physiological responses) with boredom. Moreover, our study finds that certain aspects of anticipated negative mood manifest as boredom symptoms: an increase in pressure and stress levels related to exams or academic projects; problems arising from the personal life of each athlete; or physical fatigue after intense periods of training. Findings from Chin et al. (2017) support our findings from this category as boredom is more likely to co-occur with negative emotions. Sports managers and coaches must show a more personal and direct involvement with athletes. Emotional intelligence is a relevant skill to develop in both sports managers and coaches. It seems valuable to follow each athlete on an individual basis and to identify how they feel emotionally at the various stages of the periodic training cycle to prevent emotional imbalances that may affect their performance and diet.

Lack of motivation or seriousness among peers is another psychological determinant of boredom in athletes. The third category links boredom in athletes with their perceptions that their teammates do not show commitment. Involvement level in a task refers to how much importance a person assigns to a task and how much they identify psychologically with that task (Lodahl and Kejnar, 1965). Personal involvement in a task is construed as a motivational factor within the social system of an organization (i.e., professional, amateur, and college teams) in which social norms and values (e.g., lack of seriousness and involvement) play an important role (Dubin and Dubin, 1974; Carter, 2017). Furthermore, Vodanovich and Watt (2016) proposed that boredom proneness is negatively correlated with task involvement. In order to avoid this situation, it is very important that coaches have the ability to identify boredom among athletes. On the other hand, coaches must decide if athletes who show a lack of commitment should leave the team, as it seems that boredom becomes toxic and contagious to the rest of the teammates.

The fourth category is representative of the relationship between waiting periods and boredom in athletes. In situations where people must wait, boredom occurs (Darden, 1999). Wait-related boredom generates stress and dissatisfaction (Pruyn and Smidts, 1998), enables meta-cognition systems as people allocate additional cognitive resources, and demonstrates self-monitoring behaviors into prospective timing (Zakay, 2014), and results in contextual assessments of the situation to identify the causes of boredom (Van den Bergh and Vrana, 1998). The waiting time between exercises is a critical point in the planning of training activities because it is a major reason why athletes feel bored. Generally, training plans focus on the exercises to be performed, but they do not take into account what happens to athletes during periods of waiting and during transitions from one exercise to another. Garn et al. (2017) proposed that, in physical education classes, waiting produces boredom. Athletes in our sample confirmed this argument. Coaches should consider assigning sub-activities or dynamic games to combat boredom during these waiting and transitional periods.

The absence of declared objectives or lack of competition is an aspect that significantly affects the levels of boredom in athletes. The fifth category shows that athletes’ episodes of feeling bored are connected to their perceptions that they are not being challenged enough. When athletes perceive that there are no forthcoming competitions, coaches do not set goals, and they are not being challenged by teammates or other athletes, they feel bored. Previous research by Cervelló and Santos-Rosa (2001) revealed that there is a positive correlation between boredom and athletes being competitive and ego-oriented. Perceptions of challenges, goal orientation, and competitiveness in athletes are influenced by the nature of social relationships with their colleagues and teammates (Smith et al., 2006). This is evident in athletes’ responses. It is a challenge for coaches to achieve a high level of motivation in their athletes when the athletes feel that there is no goal to achieve in the near future. It is recommended that coaches and team managers plan an unofficial tournament schedule to maintain the motivation of athletes in periods when there is no official competition. If sports competitions follow a seasonality variance, sports managers need to find new ways to challenge athletes.

Lack of participation in training activities also causes boredom in athletes. In line with category six findings, Velasco (2017) reported that boredom is a consequence of people perceiving their tasks as insignificant or artless, and it occurs in situations where specific skills are not necessary. Additionally, when individuals subjectively perceive that they have too much time available or too little to do, they engage in leisure boredom (see Iso-Ahola and Weissinger, 1990). As this category validates, leisure boredom in athletes happens as a consequence of them perceiving they have not reached an optimal discretionary time for what they consider “productive” sports activities. Although the level of incidence of this category is relatively low, sports managers must also pay attention to specific tasks that generate boredom. For example, concentrating athletes’ work on auxiliary activities during training sessions, such as picking up balls, setting up the field, preparing the training equipment, and other similar actions necessary to carry out practices, can cause a lack of motivation and engagement, thus triggering boredom. Therefore, it is necessary for coaches to plan auxiliary activities to be carried out by all players proportionally in order to maintain a sense of fairness and involvement within the group.

The lack of empathy with coaches and teammates is also a social aspect to consider. Category seven reflects that when athletes establish social distance with others, or simply develop interpersonal conflict, feeling of boredom appear. Evidence from Van Tilburg and Igou (2011) suggested that boredom increases affiliation with the in-group (i.e., like-minded teammates) as well as psychological distance from the out-group (i.e., a new coach or a teammate who others consider too competitive). In any work environment it is necessary for the group leaders to make an effort to improve interpersonal relationships and increase harmony among the team members. In the realm of sports management, coaches are the ones to carry out this work, as they fulfill the role of formal group leaders. To foster empathy, it is recommended that coaches and athletes engage in leisure and recreation activities outside the field related to their sport discipline.

The last category involves boredom incidents in relation to sports infrastructure issues. Since the *servicescape* produces cognitive, affective, and physiological responses in both employees (i.e., coaches) and customers (i.e., athletes; Bitner, 1992), it is not surprising that issues arising from the *sportscape* produce boredom. Since this category indicates facilities maintenance and sports equipment can have this effect, sports managers need to plan budgets to address infrastructural issues.

### Boredom Predicting Athletes’ Performance, Brand Preferences, and Overconsumption Behaviors

This research also provides evidence that boredom can influence athletes’ brand preferences and overconsumption behaviors. Boredom-prone athletes are more susceptible to decreases in their performance, are more inclined to vary their brand preferences (demonstrating variety seeking), and engage in impulse buying by purchasing additional products even when they do not need them.

Study 2 identifies that boredom negatively affects athlete’s performance. In sum, the higher the level of boredom proneness in athletes, the more likely it is that their performance will be diminished. This result is in line with previous organizational psychology research identifying that boredom diminishes productivity, task engagement, and performance (Drory, 1982; Kass et al., 2001; Watt and Hargis, 2010; Harju et al., 2014; Wan et al., 2014). Among the control variables included in the first regression model, only age demonstrates a positive and statistically significant effect on athletes’ performance. Thus, older elite athletes perform better than younger ones.

Findings from Study 2 also highlight that athletes prone to feeling bored engage in variety-seeking behaviors, as they demonstrate preferences for a greater number of sport drink brands. Past research shows that variety-seeking behaviors can manifest in brand preference (Trijp et al., 1996). Zandstra et al. (2004) advised that consumers might change their *repertoire* of food purchases due to boredom. Ha and Jang (2013) found evidence that boredom leads to variety-seeking behaviors in food choices. And Steenkamp and Baumgartner (1992) suggested that variety seeking provides relief from feeling bored. Thus, our results are in accordance with previous boredom literature.

In addition to the abovementioned findings, Study 2 provides evidence that boredom-prone athletes engage in overconsumption behaviors. In the presence of sales promotions, athletes with higher boredom proneness are inclined to purchase more products when they do not need them. Previous studies describe that when consumers feel bored, they seek rewards and engage in impulse buying to avoid their negative mood (Gardner and Rook, 1988). This phenomenon is common in environments like online shopping (Sundström et al., 2019), at airports when traveling (Crawford and Melewar, 2003), and in retail shopping (Sharma et al., 2010).

In the next section, for each of the categories of boredom, we have analyzed the level of incidence and discuss the implications for sports management.

### Other Implications for Sports Management

It is evident in the study that boredom shapes athletes’ buying behaviors. If athletes are boredom prone, they might alter their strict diets and make unhealthy, reward-seeking choices (e.g., consuming alcohol, drugs, high-level sugar products, etc.). Additionally, when boredom exists, impulse buying behavior and conspicuous consumption happen. Since boredom leads to a greater search for stimulation, athletes could engage in overconsumption. Therefore, it is recommended that coaches observe the consumption patterns of their athletes to prevent situations that could affect their emotional balance or even their personal finances and health.

Interestingly, applying CIT to both sets of data showed that sources of boredom for coaches and athletes fall into similar categories. Appendix B in the Supplementary Material illustrates the categories associated with boredom incidents, including lack of athletes’ motivation or involvement (35.3% of the total incidents), monotonous or repetitive tasks (29.4%), anticipated negative mood (17.6%), and lack of competitions and forthcoming tournaments/goals (11.8%). Although the sample size is small, this exercise replicates our findings and extends the generalizability of the categories related to boredom incidents.

We interviewed coaches who train elite athletes to get their perspectives on how sports management can help them cope with athletes’ boredom. This feedback helped us identify the ways that sports managers can reduce the impact of athletes’ boredom (e.g., by using technological and infrastructure resources; administration of inter-group relationships; and motivational factors, planning skills, and other capabilities). Coaches rely on new technologies, materials, and equipment as institutional resources to reduce episodes of boredom in athletes. Some of these feedback items suggest that creating a sense of novelty and interacting with technology can help in the quest to reduce boredom. Additionally, coaches refer to their need to develop social skills (i.e., inter-group relationships) and introduce motivational factors to cope with athletes’ boredom. The role of sports management in both dimensions is crucial. Coaches identify that management needs to be in constant contact with athletes to motivate them. Moreover, creative solutions, such as inviting the press to cover athletes’ stories, spending more money for teams to participate in competitions, and simply letting players play music at practices, serve as tools to reduce boredom incidents. Finally, coaches strongly suggest that sports managers plan according to the type of teams and players they manage. Planning skills in terms of designing practices are relevant. Table 4 provides a summary of coaches’ feedback on how to deal with athletes’ boredom episodes.

**TABLE 4:** Coaches feedback on how to deal with athletes’ boredom.

Institutional capabilities	Illustrative quotes from coaches on how to cope with athletes’ boredom
1. Technology/infrastructure	The team needs new materials like videos and analytics software.**_soccer and weightlifting coaches_**
	We need new equipment that fulfills safety standards for athletes to practice more difficult and risky exercises.**_gymnastics coach_**
	I have asked several times not to use the coliseum for other events that are not sport related.**_basketball coach_**
	A game room.**_track and field coach_**
	New equipment.**_all coaches_**
2. Inter-group relationships	I need to develop skills in integrating players, motivating them, and making them all to participate in our practices.**_ men’s soccer coach_**
	More presence at practices of the management team in order for players to perceive their interest and support.**_ taekwondo coach_**
	We should have a social event.**_ women’s soccer coach_**
3. Motivational factors (e.g., competitions)	For me, it is necessary to invite the press to our facilities for them to cover our stories.**_jiu–jitsu coach_**
	Increase the number of scholarships or fellowships due to athletes’ high performance. **_basketball coach_**
	Playing music at practices using selected playlists.**_volleyball coach_**
	Register our team in additional national or international tournaments.**_jiu–jitsuandtenniscoach_**
4. Planning	I would like to spend more time with the high-end athletes that compete for our university than coaching non-experienced teams.**_gymnastics coach_**
	It will be great to consider a pause in the activities, but we cannot stop the rhythm.**_aerobics instructor_**
	Funding availability to register in conferences, symposia, and training.**_triathlon coach_**
5. Other	I bet the players will like to listen to other experienced players, so guest speakers might work.**_jiu–jitsu coach_**

*^1^For a review about the CIT check Butterfield et al. (2005); to check its appropriateness to examine human emotions see Buckley (2016); and to check how the CIT was previously utilized to study boredom see Velasco (2017).*

### Limitations and Future Research

The results of this research should be seen in the light of some limitations. First of all, our study had a cross-sectional design, which restricted our ability to generalize the findings. Second, we had decided to use a single-item measure to capture athletes’ performance. Because each coach who participated in this study uses different performance indicators, it was difficult for us to extrapolate additional items to evaluate athletes’ performance. However, the measure we used was a result of a joint effort between the coaches and the authors to develop a measure that is capable of identifying both the top performers and the irregular performers. Third, we used a short version of the boredom proneness scale due to the time limitations of our sample of athletes. Future studies should use longer versions of the boredom proneness scales, such as the ones developed by Farmer and Sundberg (1986) and Vodanovich et al. (2005), as a way to test the reliability of our results. In addition, the findings from Study 2 might need careful consideration as we unfortunately did not control for mood when examining the relationships among the variables included in our regression models. Finally, one individual included in our sample is a 66-year-old triathlon athlete. Evidence from Vondanovich and Kass (1990) shows that age influences boredom, as older individuals are ought to be less prone to boredom. That is why age was included as a control variable in our regression models.

Finally, taking into consideration our findings, we suggest a venue of topics for future studies that will allow for a greater understanding of the prevalence of boredom in athletes and its effects. In particular, these recommendations include further investigation of the role of boredom in athletes’ nutritional diets, online shopping behaviors, and advertising persuasiveness.

## Data Availability Statement

The datasets generated for this study are available on request to the corresponding author.

## Ethics Statement

The studies involving human participants were reviewed and approved by the USFQ Comité de Ética. The patients/participants provided their written informed consent to participate in this study.

## Author Contributions

Both authors contributed equally to the investigation. Content analysis of boredom incidents was equality distributed among the authors and independent judges. In the data collection process, a team of research assistants helped the authors with the interviews and the survey data collection.

## Conflict of Interest

The authors declare that the research was conducted in the absence of any commercial or financial relationships that could be construed as a potential conflict of interest.
