# Supplementary material for: Portrait of Boredom Among Athletes and Its Implications in Sports Management: A Multi-Method Approach
Source: Front Psychol. 2020 May 26;11:831. doi: 10.3389/fpsyg.2020.00831 (PMC7264414; doi:10.3389/fpsyg.2020.00831)
Supplement: Supplementary file 1 [file Data_Sheet_1.docx]

**Appendix A – supplementary material-**

**Factor Loadings for Athletes’ Boredom Proneness Scale**

| Item | Question | Loading |
| --- | --- | --- |
| 1 | - It is difficult for me to concentrate on my training activities | 0.398 |
| 2 | Time always passes very slowly when I attend practices | 0.791 |
| 3 | Many things I have to do during practices are repetitive and monotonous | 0.795 |
| 4 | It takes more stimulation to get me going than most players” | 0.585 |
| 5 | At practices, I am bad at waiting patiently | 0.389 |

KMO index for sampling adequacy = 0.629; *x*^2^ = 74.089, p< 0.001

**Appendix B –supplementary material --**

**Percentage of Coaches’ Boredom Incidents Falling into each Content Category**

| **Category Name** | **Illustrative quotes** | **Percentage of Total** |
| --- | --- | --- |
| - 1 Athletes’ lack of motivation or involvement | I feel bored when my best performance athletes fail in their fundamentals and I have to go back to basics with them*.* **_weightlifting coach_**  A sense of disinterest and apathy of players at practice sessions in which a large number of interruptions exist to amend the players’ mood. **_basketball coach_**  When I have to escort a group of amateur cyclists that care more about sightseeing than improving their performance. **_triathlon coach_**  I carefully planned my practice sessions in a sequence and with a specific number of players, but they came late or did not show up. **_basketball coach_** | **35.3%** |
| 2 Monotonous or repetitive tasks | I am terribly bored because I have been coaching novices for so many years and using the same training exercises*.* **_jiu-jitsu coach_**  When the training session includes repetitive activities like practicing formations and dump shots. **_volleyball coach_**  It normally happens at the last 10 to 15 minutes of my late afternoon classes, after teaching all morning*.* **_aerobics instructor_** | **29.4%** |
| 3 Anticipated negative mood | I was feeling irritated at practices because I still play at competitive level and practices became boring to me. **_Soccer coach_**  When I start having pessimistic type of thoughts, I feel bored. For example, I start imagining we might lose the next game. **_Soccer coach_** | **17.6%** |
| 4 Not having forthcoming competitions, challenges, or goals | There are no so many tournaments in the year, so I start feeling bored when I have to work on administrative tasks. Like preparing reports and evaluating the performance of my players.  **_tennis table coach_**  There are those days that I have to improvise in the practice design, and I start feeling bored as there is no specific goal and players notice that. **_tennis coach_** | **11.8%** |
| 5 No boredom episodes reported/described | N/A | **5.9%** |
